# High coverage of diverse invasive meningococcal serogroup B strains by the 4-component vaccine 4CMenB in Australia, 2007–2011: Concordant predictions between MATS and genetic MATS

**DOI:** 10.1080/21645515.2021.1904758

**Published:** 2021-04-13

**Authors:** Sarah J. Tozer, Helen V. Smith, David M. Whiley, Ray Borrow, Giuseppe Boccadifuoco, Duccio Medini, Davide Serruto, Marzia Monica Giuliani, Maria Stella, Rosita De Paola, Alessandro Muzzi, Mariagrazia Pizza, Theo P. Sloots, Michael D. Nissen

**Affiliations:** a Queensland Paediatric Infectious Disease Laboratory, Children’s Health Queensland Hospitals and Health Service, Queensland Children’s Hospital, Brisbane, Australia; b Child Health Research Centre, The University of Queensland, Brisbane, Australia; c Pathology Queensland, Forensic & Scientific Services, Brisbane, Australia; d Public Health England, Meningococcal Reference Unit, Manchester Royal Infirmary, Manchester, United Kingdom; e GSK, Siena, Italy; f GSK, Melbourne, Australia

**Keywords:** Meningococcal serogroup B, 4CMenB, MATS, gMATS, strain coverage, Australia

## Abstract

Meningococcal serogroup B (MenB) accounts for an important proportion of invasive meningococcal disease (IMD). The 4-component vaccine against MenB (4CMenB) is composed of factor H binding protein (fHbp), neisserial heparin-binding antigen (NHBA), *Neisseria* adhesin A (NadA), and outer membrane vesicles of the New Zealand strain with Porin 1.4. A meningococcal antigen typing system (MATS) and a fully genomic approach, genetic MATS (gMATS), were developed to predict coverage of MenB strains by 4CMenB. We characterized 520 MenB invasive disease isolates collected over a 5-year period (January 2007–December 2011) from all Australian states/territories by multilocus sequence typing and estimated strain coverage by 4CMenB. The clonal complexes most frequently identified were ST-41/44 CC/Lineage 3 (39.4%) and ST-32 CC/ET-5 CC (23.7%). The overall MATS predicted coverage was 74.6% (95% coverage interval: 61.1%–85.6%). The overall gMATS prediction was 81.0% (lower–upper limit: 75.0–86.9%), showing 91.5% accuracy compared with MATS. Overall, 23.7% and 13.1% (MATS) and 26.0% and 14.0% (gMATS) of isolates were covered by at least 2 and 3 vaccine antigens, respectively, with fHbp and NHBA contributing the most to coverage. When stratified by year of isolate collection, state/territory and age group, MATS and gMATS strain coverage predictions were consistent across all strata. The high coverage predicted by MATS and gMATS indicates that 4CMenB vaccination may have an impact on the burden of MenB-caused IMD in Australia. gMATS can be used in the future to monitor variations in 4CMenB strain coverage over time and geographical areas even for non-culture confirmed IMD cases.

## Introduction

Invasive meningococcal disease (IMD), caused by *Neisseria meningitidis*, is a rare but life-threatening condition which remains a substantial health concern. The incidence of IMD is relatively low, ranging from 0.01 to 3.6 annual cases/100,000 persons across different geographic and socio-economic settings worldwide over the last decade.^1^ The epidemiology of IMD is also diverse, with 6 meningococcal serogroups (MenA, MenB, MenC, MenW, MenY, and MenX) causing the majority of the disease, but it varies geographically and especially over time, following unpredictable dynamics. MenB is currently the prominent serogroup causing IMD in Australia, Europe, part of Africa and the Americas.^1^

In Australia, MenB was the predominant serogroup causing IMD (≥50% of cases), between 2001 and 2015, despite a progressive decline in incidence (from 1.52 to 0.47/100,000 population).^2,3^ After an increase and subsequent decrease in the proportion of MenW and MenY cases during 2016–2017, MenB accounted for 52% of IMD cases in the first half of 2019, when the overall incidence of IMD was 0.4/100,000.^4^ MenC vaccination was introduced in the national immunization program in 2003 (as a single dose at 12 months of age and catch-up campaigns targeting 2–19-year-olds) and resulted in a decline of 96% in the incidence of MenC-caused disease within 10 years.^5^ Starting with 2019, MenC has been replaced by MenACWY vaccination in all Australian states and territories.^6^

The development of an efficacious vaccine against MenB has been limited by the poor immunogenicity of the MenB capsular polysaccharide. Currently, 2 protein-based vaccines against MenB, a 4-component MenB vaccine (*Bexsero*, GSK; 4CMenB) and a bivalent recombinant factor H binding protein (fHbp) vaccine (*Trumenba*, Pfizer; rLP2086) are available for use in adolescents in several countries worldwide. 4CMenB is the only MenB vaccine licensed for use in infants. 4CMenB includes 3 novel genome-derived antigens, namely fHbp, *Neisseria* adhesin A (NadA), neisserial heparin-binding antigen (NHBA), as well as outer membrane vesicles of a New Zealand MenB strain expressing porin A (PorA) 1.4 as the major antigen.^7^

Due to the dynamic genotypic and phenotypic variability between and within MenB strains,^8–10^ it is difficult to evaluate the degree of protection offered by a vaccine, as it may vary from one strain to another. For licensure purposes, vaccine efficacy for 4CMenB was inferred from immunogenicity data: a serum bactericidal antibody assay with human complement (hSBA) titer ≥1:4 against 4 reference strains, specific to the vaccine antigens, was considered as indicative of protection. Testing serum bactericidal activity across all circulating strains is impractical, if not impossible, and this is especially true for infants, as serum volumes collected from them are limited. As an alternative to hSBA testing, the meningococcal antigen typing system (MATS) was developed to evaluate coverage of 4CMenB across circulating strains. MATS combines a unique vaccine antigen-specific sandwich enzyme-linked immunosorbent assay (ELISA), which measures both immunological cross-reactivity and relative quantity for 3 recombinant antigens, with PorA genotyping information for the outer membrane vesicle component,^11,12^ and was shown to be a conservative predictor of serum bactericidal activity and individual protection.^13,14^ A complementary method, the genetic MATS (gMATS), based on the correlation between vaccine antigen genotyping and MATS data, has also been developed and employed to assess potential coverage of 4CMenB across MenB strains.^15^

**Figure 1.**
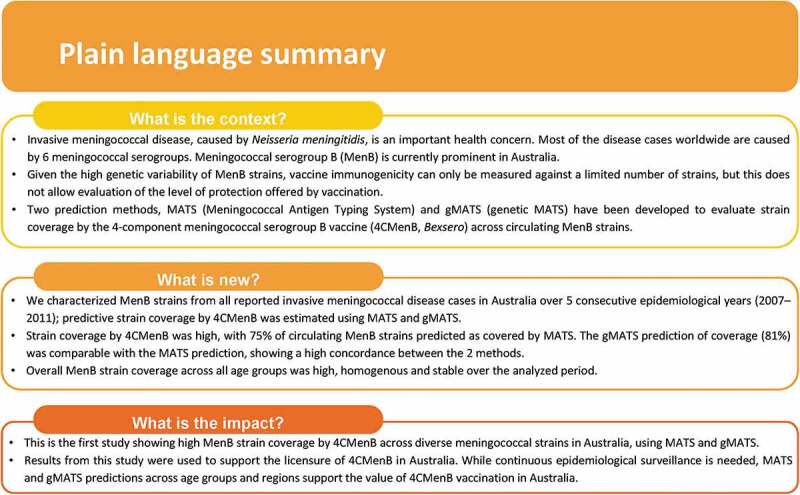
Plain language summary

Here, we characterized by multilocus sequence typing (MLST) and vaccine antigen sequencing/genotyping a panel of 520 invasive MenB strains isolated in Australia during 5 consecutive epidemiological years (from 2007 to 2011) and evaluated coverage of 4CMenB by MATS and gMATS (Figure 1).

## Methods

### Meningococcal strains tested

A total of 520 MenB isolates obtained from all reported Australian clinical cases of IMD between January 2007 to December 2011 were tested in this study. These were collected by the National *Neisseria* Network (NNN) State Reference Laboratories throughout all states and territories in Australia. In total, 156 strains were collected from New South Wales/Australian Capital Territory, 141 from Queensland, 118 from Victoria, 52 from Western Australia, 40 from South Australia, 9 from Tasmania, and 4 from Northern Territory. The clinical presentations in patients from whom the samples were collected were primarily meningitis and septicemia.

### MLST and antigen genotyping

MLST was performed as previously described.^16^ The MenB isolates were profiled by sequencing the *abcZ, adk, aroE, fumC, gdh, pdhC* and *pgm* genes. Sequence data were analyzed and compared against the PubMLST *Neisseria* MLST database (https://pubmlst.org/organisms/neisseria-spp) to determine the sequence type (ST) and clonal complex (CC) for each isolate. Genotyping of vaccine antigens was also performed for the entire panel of strains by polymerase-chain reaction (PCR) and sequencing using published methods.^15,17^

### MATS testing

MATS testing was performed at Public Health England and/or the Queensland Health laboratories following a standardized protocol. In Australia, bacterial growth and preparation of bacterial cell extracts was performed at the Communicable Diseases Unit at Forensic and Scientific Services, Coopers Plains, Queensland, and MATS ELISA was performed at the Queensland Pediatric Infectious Disease Laboratory, Queensland Children’s Hospital, Children’s Health Service, South Brisbane, Queensland.

Levels of expression and cross-reactivity for fHbp, NadA, and NHBA were analyzed by the MATS ELISA, PorA serosubtype was determined, and strain coverage for each vaccine component was assessed as previously described.^11^ Briefly, for each of the fHbp, NadA, and NHBA vaccine antigens, the MATS ELISA was expressed as the relative potency (RP) of the tested strain compared to a reference strain. The RP value determines the prediction for the strain as covered or not covered depending on whether it is higher or lower than a positive bactericidal threshold (PBT), defined as the minimum RP predicting bactericidal killing of the strain in hSBA. Strains for which RP** **>** **PBT for one or more vaccine antigens were considered as covered. Coverage by the PorA component was estimated by sequencing the fragment of the *porA* gene encoding variable region 2 (VR2): strains expressing PorA serosubtype 1.4 (VR2 = 4) were considered as covered.^18^

Coverage by 4CMenB was defined as the percentage of strains covered by at least one vaccine antigen, using PBT thresholds values of 0.021 (95% coverage interval [CI]: 0.014–0.031), 0.294 (95% CI: 0.169–0.511), and 0.009 (95% CI: 0.004–0.019) for fHbp, NHBA, and NadA, respectively.^11,19^ CIs were estimated based on MATS precision (19.8% for fHbp, 28.8% for NHBA, and 38.3% for NadA), as determined during an inter-laboratory standardization.^19^

### gMATS

The potential coverage of 4CMenB was assessed by analyzing antigen sequences through gMATS, as previously described.^15^ The *fHbp* and *nhba* genes were PCR-amplified and sequenced, or their sequences were extracted from the whole genome sequence when available, while the *nadA* gene was PCR-amplified to determine gene presence/absence (sequencing was not performed for all isolates). Data on gMATS coverage of the present panel by antigen and antigen combination were calculated and compared with the MATS estimate. Sixteen fHbp and 9 NHBA peptides and a match for PorA VR2 peptide 4 were identified as predictors of coverage, while NadA is always considered as not contributing to coverage in gMATS.^15^

### Statistical analyses

The number of strains covered by 0, 1, 2, and/or 3 vaccine antigens was estimated by MATS and gMATS. Predictions were further stratified by year of strain collection, state/territory of collection and, where documented, by patient age group (<1 year, 1–<2 years, 2–<5 years, 5–29 years and >29 years of age).

If the number of strains predicted as covered by MATS for each stratum was sufficient to provide statistical relevance, differences between strata were calculated. Differences across strata by years of isolate collection and patient age group in the predicted MATS coverage were evaluated using the chi-square test (2-sided). Differences among states/territories were evaluated using the multiple comparisons correction (Hochberg method).

The gMATS overall predictions were compared to the MATS outcome. In particular, gMATS performance to call covered (positive) and not covered (negative) strains was evaluated by different estimators. Accuracy (fraction of true calls among all), the positive predictive value (fraction of true positive among the positive calls of gMATS), negative predictive value (fraction of true negative among the negative calls of gMATS), sensitivity (fraction of true positive calls among the positive in MATS) and specificity (fraction of true negative calls among the negative in MATS) were assessed on the subset of strains predictable by gMATS. By their definitions, sensitivity and specificity are interpreted as the likelihood to correctly predict original positive and negative MATS results. Positive and negative predictive values as the likelihood that predicted positive and negative by gMATS are true.

Analyses were performed using the R package software.

## Results

### MLST and antigen sequencing/genotyping

**Figure 2.**
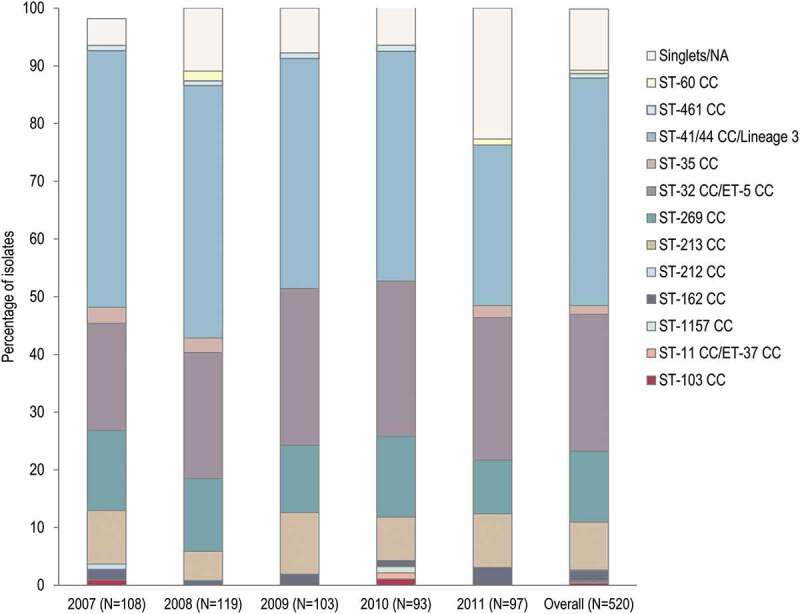
Clonal complexes distribution in the 520-strain panel, by year and overall

NA, genotyping data not available; ST, sequence type; CC, clonal complex; ET, electrophoretic type; N, number of isolates.

In total, 12 CCs were identified, with 4 of them including 435 (83.7%) strains: ST-41/44 CC/Lineage 3 (39.4%), ST-32 CC/ET-5 CC (23.7%), ST-269 CC (12.3%) and ST-213 CC (8.3%). No major differences were observed in the distribution of CCs between the different years (Figure 2). The most prominent identified ST was ST-32, represented by 55 (10.6%) strains. Of the 520 isolates, 55 (10.6%) were classified as singlets.

**Figure 3.**
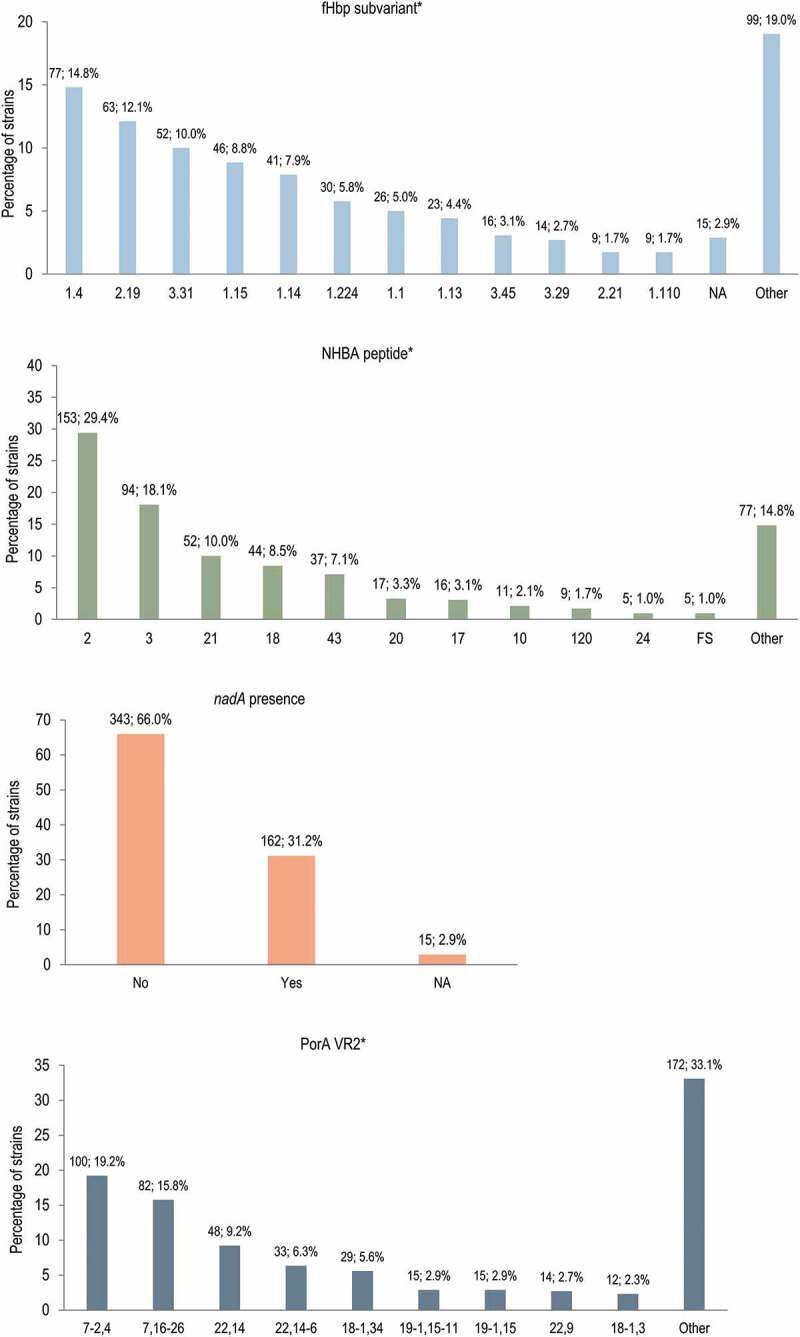
4CMenB peptides and subvariants and their distribution in the 520-strain panel

Antigen sequence characterization showed that fHbp variant 1 was the most prevalent (in 313 [60.2%] isolates), while variants 2 and 3 were present in 106 (20.4%) and 100 (19.2%) isolates. The fHbp subvariants 1.4, 2.19, and 3.31 were the most common among the isolates (Figure 3). For technical reasons, the sequencing of 15 strains was incomplete, and hence the whole gene sequence was not available. The NHBA peptide 2, which is included in 4CMenB, was detected in 153 (29.4%) isolates, and peptide 3 was the second most frequently detected (94 [18.1%] isolates). The *nadA* gene was present in 162 (31.2%) isolates, while for 15 isolates *nadA* presence/absence could not be confirmed because the PCR amplification step anchored to the gene flanking region was negative. For porA, the most prevalent (VR1, VR2) profiles were 7-2,4 in 100 (19.2%) isolates, and 7,16-26 in 82 (15.8%) isolates. The PorA 1.4 subtype, which is an exact match for the vaccine variant, was detected in 105 (20.2%) isolates.

4CMenB, 4-component meningococcal serogroup B vaccine; fHbp, factor H binding protein; NHBA, neisserial heparin binding antigen; *nadA, Neisseria* adhesin A; PorA, porin A; VR, variable region.Notes: the labels above the bars indicate the number and percentage of strains with the same genotype feature. * Only peptides/profiles detected in ≥2% of isolates are shown.

### Predicted vaccine coverage

Overall, MATS analysis estimated that 74.6% (95% CI: 61.1–85.6) of the 520-strain panel would be covered by 4CMenB. The predicted gMATS coverage was 81.0% (lower limit [LL] – upper limit [UL]: 75.0–86.9) (Table 1). Overall, 62 (11.9%) strains were unpredictable by gMATS, including 9 (1.7%) strains for which complete sequencing/genotyping data was not available (Supplementary Table 1). The overall gMATS prediction showed an accuracy of 91.5% compared to MATS. Sensitivity, specificity, and the positive and negative predictive values were 98.1%, 65.6%, 91.8% and 89.7%, respectively.

**Table 1.** Strain coverage predicted by MATS and gMATS, overall and by year of sample collection, state/territory, and the patient’s age group

	N	MATS coverage, % (95% CI)	gMATS coverage, % (LL-UL)
**Overall**	**520**	**74.6 (61.1–85.6)**	**81.0 (75.0–86.9)**
**Byyear**			
2007	108	70.4 (57.4–83.3)	79.2 (74.1–84.3)
2008	119	81.5 (69.7–89.0)	82.8 (76.5–89.1)
2009	103	70.9 (58.2–81.5)	80.1 (75.7–84.5)
2010	93	77.4 (60.2–87.1)	87.6 (81.7–93.5)
2011	97	72.2 (58.8–86.6)	75.3 (67.0–83.5)
**By state/territory**			
New South Wales & ACT	156	71.8 (54.5–82.0)	78.5 (71.8–85.3)
Northern Territory	4	–*	62.5 (50.0–75.0)
Queensland	141	85.1 (73.0–94.3)	87.2 (84.4–90.1)
South Australia	40	90.0 (90.0–95.0)	93.8 (92.5–95.0)
Tasmania	9	–*	66.7 (66.7–66.7)
Victoria	118	73.7 (56.8–85.6)	80.9 (71.2–90.7)
Western Australia	52	51.9 (42.3–71.1)	65.4 (57.7–73.1)
By age group**			
<1 year	75	64.0 (53.3–74.7)	68.7 (58.7–78.7)
1–<2 years	28	71.4 (64.3–89.3)	76.8 (67.9–85.7)
2–<5 years	30	83.3 (66.7–90.0)	98.3 (96.7–100)
5–29 years	118	80.5 (66.1–91.5)	86.4 (82.2–90.7)
>29 years	68	75.0 (58.8–86.8)	77.9 (72.1–83.8)

MATS, meningococcal antigen typing system; gMATS, genetic MATS; 4CMenB, 4-component meningococcal serogroup B vaccine; N, number of tested isolates; CI, coverage interval; LL, lower limit; UL, upper limit; ACT, Australian Capital Territory.

Notes: * Not calculated, due to the small number of strains isolated.

** The age of the patient from whom the sample was collected was only documented for 319 of the total 520 strains.

**Figure 4.**
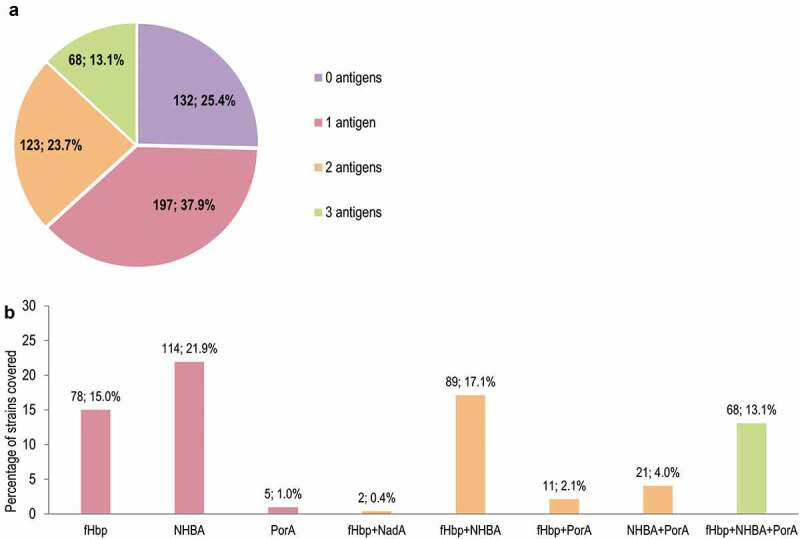
Number and percentage of MenB strains from the 520-strain panel covered in MATS, by number of 4CMenB vaccine antigens (a) and vaccine antigen/combination of antigens (b)

MenB, meningococcal serogroup B; MATS, meningococcal antigen typing system; 4CMenB, 4-component MenB vaccine; fHbp, factor H binding protein; NHBA, neisserial heparin binding antigen; NadA, *Neisseria* adhesin A; PorA, porin A. Note: the labels above the bars indicate the number and percentage of covered strains.

**Figure 5.**
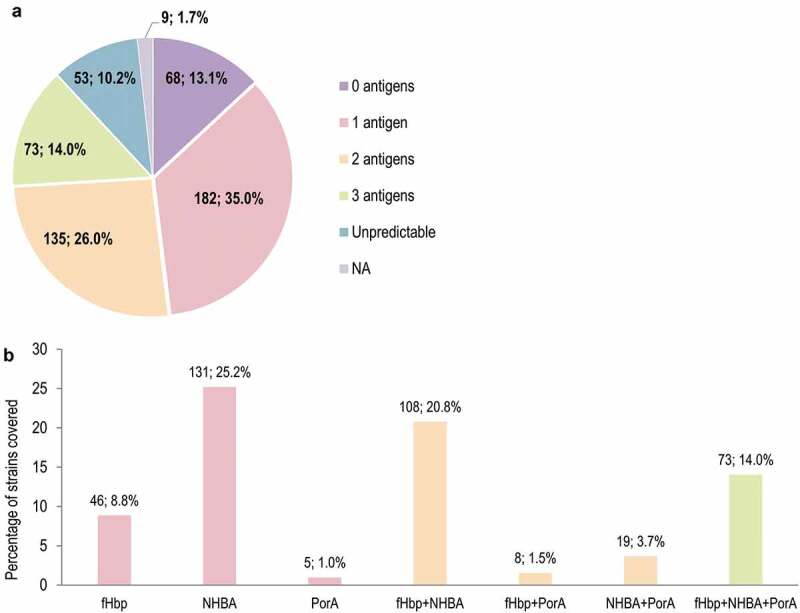
Number and percentage of MenB strains from the 520-strain panel covered in gMATS, by number of 4CMenB vaccine antigens (a) and vaccine antigen/combination of antigens (b)

MenB, meningococcal serogroup B; gMATS, genetic meningococcal antigen typing system; 4CMenB, 4-component MenB vaccine; NA, genotyping data not available; fHbp, factor H binding protein; NHBA, neisserial heparin binding antigen; PorA, porin A. Note: the labels above the bars indicate the number and percentage of covered strains.

The proportions of strains covered in MATS by one or more antigens is shown in Figure 4a. The antigen contributing most to MATS coverage was NHBA (292 [56.2%] strains predicted to be covered by NHBA alone or in combination with other antigens), followed by fHbp (248, 47.7%), PorA (105, 20.2%), and NadA (2, 0.4%). Overall, 68 (13.1%) strains were predicted to be covered by 3 antigens (porA+fHbp+NHBA) (Figure 4b). The proportions of strains covered by one or more antigens in gMATS are shown in Figure 5a. Of the 520-strain panel, 331 (63.7%), 235 (45.2%), and 105 (20.2%) strains were covered in gMATS by NHBA, fHbp, and PorA, respectively, alone or in combination. Seventy-three (14.0%) strains were covered by the combination of 3 antigens (porA+fHbp+NHBA) (Figure 5b). Most strains belonging to ST-41/44 CC/Lineage 3 (86.3%; LL–UL: 82.4–90.2) and ST-32 CC/ET-5 CC (91.9%; LL–UL: 88.6–95.1) were predicted to be covered by gMATS.

Data on strains covered by 0, 1, 2 or 3 vaccine antigens stratified by year of sample collection, state/territory, and patient’s age group are summarized in Supplementary Table 1 for both MATS and gMATS.

Over the 5-year period, predicted MATS yearly coverage ranged from 70.4% to 81.5% (Table 1). A comparison of point estimates of strain coverage did not identify any significant difference across years (*p*-value = 0.242). Yearly gMATS predictions ranged from 79.2% to 87.6%.

MATS coverage estimates varied by state of origin for the strains, with the highest value in South Australia (90.0%) (Table 1). For Northern Territory and Tasmania, 4CMenB coverage could not be estimated due to the low number of samples collected. A comparison between the 5 states with the highest numbers of covered isolates revealed a statistically significant difference in the predicted strain coverage between Western Australia and Queensland, and between Western Australia and South Australia (*p*-values: 4.03 × 10^−5^ and 0.002, respectively). Accordingly, the highest coverage by gMATS was predicted for South Australia (93.8%) and the lowest in the Northern Territory (62.5%).

MATS coverage by age group varied between 64.0% (for <1-year-olds) and 83.3% (for the 2–<5 years age group) (Table 1), with no significant difference across age groups (*p*-value = 0.09). gMATS coverage predictions varied by age group, from 68.7% in <1-year-olds to 98.3% in children 2–<5 years of age.

## Discussion

MATS and gMATS are established systems to predict the potential strain coverage by 4CMenB, thus helping to anticipate the public health impact of vaccination and to support post-implementation surveillance. gMATS was introduced more recently^15^ to achieve the same objectives as MATS, with 2 distinctive advantages: faster execution (wet-lab free methodology) and expanded applicability, as it can also be performed in the absence of a bacterial isolate.^20^ This is the first study to assess 4CMenB strain coverage in Australia, in a panel of 520 IMD-causing MenB isolates, using both MATS and gMATS and characterizing the strain panel through MLST and antigen sequencing.

Twelve different MenB CCs circulated in Australia during 2007–2011, with 4 of them representing the majority of isolates (83.7%). Overall, 39.4% of strains belonged to ST-41/44 CC/Lineage 3, making it the most prominent CC circulating in Australia during this period. This CC was also observed in other countries (such as the United States,^21^ Chile,^22^ England and Wales, France, Germany, Norway, Italy^23^ and the Republic of Ireland^24^) during a similar epidemiological period as that analyzed in the current study. MenB strains circulating in Australia continued to show considerable genetic diversity, with more than 10 CCs identified in 2018 and a cluster of CC41/44 (ST-154) strains showing an exact match with at least 1 of 4CMenB antigen.^25^ Among the 64 MenB Australian isolates collected between 2012 and 2019, reported in the PubMLST database and for which CC was assigned, 50% belonged to ST-41/44,^26^ showing continuous predominance of this CC in Australia.

The MATS strain coverage estimated for 4CMenB during 2007–2011 was high, with 74.6% of circulating MenB strains predicted as covered. Previously, MATS coverage predictions ranging from 66% to 91% were reported for 4CMenB across 16 countries, over different periods of time.^12,15,21,23,27–32^ The strain coverage estimated for Australia compares well with values predicted by MATS for 4CMenB in England and Wales over similar periods of time: (73%) in 2007–2008^23^ and 2015–2016,^15^ and the Czech Republic (74%), in 2007–2010.^23^

The gMATS prediction of coverage (81.0%) was comparable with the MATS prediction, with 91.5% concordance between the 2 estimates and only 11.9% of strains in the overall panel not being predictable by gMATS, also including strains with incomplete sequencing/genotyping data. Both in MATS and gMATS, NHBA, followed by fHbp, were the antigens with the most important contribution to coverage and ≥1/3 of strains were predicted to be covered by at least 2 antigens. NHBA and fHbp were also previously reported as the most important contributors to 4CMenB strain coverage in panels of strains collected from England and Wales,^23,28^ Italy,^23^ and the Republic of Ireland,^24^ where 4CMenB vaccination has been since introduced in the national immunization program.

Some differences in 4CMenB coverage by both MATS and gMATS between Australian states/territories were observed in the current analysis. However, a statistically significant difference in MATS prediction was only detected when comparing Western Australia with South Australia or Queensland. Of note, vaccine coverage estimated by MATS on strains isolated in Western Australia was similar to the coverage described using the *Bexsero* antigen sequence type (BAST) scheme on the panel of strains isolated during 2008–2012 (47.1% [95% CI: 41.1–53.1%]). The same study also showed significant differences between strains circulating in Western Australia and Victoria in the same period of time, with only 9 of the 108 BAST profiles identified being common to both states.^33^

Both MATS and gMATS coverage predictions by age group were high and homogenous in the Australian panel. The highest coverage (83.3% by MATS and 98.3% by gMATS) was observed for the 2–5 years age group. Among <1-year-olds, the age group in which IMD incidence is the highest, >64.0% of isolates were predicted as covered by both methods, indicating a potentially high impact of the vaccine on the burden of IMD in infants. This coverage is similar to that predicted by MATS (63%) in the same age group, for MenB isolates from England, Wales, and Northern Ireland collected during 2014–2015,^28^ before the introduction of 4CMenB in the national immunization program.

MATS- and gMATS-predicted coverages were high and consistent throughout the 5-year period analyzed. Changes in MenB strain coverage by 4CMenB are anticipated, as the level of expression of vaccine antigens in circulating strains varies over time and across geographical areas. However, overall estimates are not expected to dramatically change over time, as shown in the case of the United Kingdom, where only a small difference in the proportion of 4CMenB antigens-covered isolates was observed between 2007–2008 and 2014–2015, with the estimated MATS coverage varying from 73% (95% CI: 57–87) to 66% (95% CI: 52–80).^28^ Moreover, the current study covered a period of 5 years, during which time MATS estimates were ≥70.4% and gMATS estimates were ≥75.3%. This confirms a limited variation over time for the 4CMenB antigens in Australia and emphasizes the importance of monitoring the epidemiology overtime. To further support this hypothesis, we have analyzed in gMATS 2 subsets of MenB strains collected in Australia and classified in the PubMLST database (as of July 2020) on the basis of genotyping data. The percentage of strains covered by 2 or 3 antigens were 39.5% for 81 strains collected up to year 2006 and 30.4% for 23 strains collected from 2012 onwards. Throughout the 5 years covered by our study, 40.0% of isolates were covered by 2 or 3 antigens in gMATS, suggesting that if mutations in one of the encoding genes could happen, the coverage estimates should not be impacted.

MATS, and by extension gMATS, underestimate bactericidal activity induced by 4CMenB in the hSBA^14,34^ and are therefore likely to underestimate vaccine coverage across MenB strains. More accurate coverage estimates can be inferred from effectiveness data, which are becoming available following the implementation of 4CMenB vaccination in the national immunization program in the United Kingdom, which started in 2015. Within 3 years from introduction of 4CMenB, administered as a 3-dose schedule at 2, 4, and 12 months of age, a 75% reduction in MenB IMD cases in infants has been observed in England.^35^ A reduction of 65% (within 5 years from introduction) and 35% (within 4 years from introduction) in MenB IMD cases was also observed in 2 regions in Italy in which routine 4CMenB vaccination was implemented according to different schedules.^36^

Partial results of this study were used to support the licensure of 4CMenB in Australia, in individuals aged ≥2 months. Since October 2018, South Australia implemented a state-funded MenB immunization program with 4CMenB, for infants 6 weeks–12 months of age, and catch-up vaccinations for children >12 months–<4 years of age. The program was expanded in 2019 to include individuals 17–21 years of age.^37^ The minimum coverage of 74.6% of the strains analyzed in this study is reassuring on the potential benefit of 4CMenB vaccination in Australia.

This study has some potential limitations in addition to those inherent to the methods used.^11,15^ The age of individuals from whom the tested sample were collected was not documented for all cases, therefore the results stratified by age should be interpreted with caution. Since MATS can only be used with cultured isolates,^11^ the prediction of strain coverage only included IMD cases for which cultured isolates were available. The intrinsic value of gMATS resides also in its ability to predict coverage for PCR-confirmed IMD cases as well,^15^ which represent an increasingly important proportion of reported cases worldwide. In Australia, around 22% of IMD cases were diagnosed using PCR alone in 2018,^24,25^ emphasizing the role of genomic approaches such as gMATS, BAST and the recently-developed Meningococcal Deduced Vaccine Antigen Reactivity (MenDeVar)^38^ in the assessment of vaccines against MenB. This study represents an additional evidence on the high concordance between MATS and gMATS predictions, as already shown on strain panels deriving from 13 countries worldwide^15^ and further supports the added value of gMATS for the prediction of 4CMenB coverage over time and across geographies.

## Conclusion

4CMenB was predicted to cover ≥74.6% of MenB strains causing IMD in Australia between 2007 and 2011, indicating that its use may reduce the burden of MenB-caused disease. In view of temporal and regional fluctuations in antigen expression and diversity observed across MenB isolates, continuous surveillance may be needed to anticipate the need for adequate vaccination strategies. The results of our study, showing a high concordance between MATS and gMATS estimates, indicate that gMATS can be used, in the future, to predict the potential impact of 4CMenB vaccination on IMD epidemiology in Australia.

## Supplementary Material

Supplemental Material

## Trademark statement

*Bexsero* is a trademark owned by or licensed to the GSK group of companies. *Trumenba* is a trademark owned by or licensed to Pfizer.
