# Supplementary material for: High coverage of diverse invasive meningococcal serogroup B strains by the 4-component vaccine 4CMenB in Australia, 2007–2011: Concordant predictions between MATS and genetic MATS
Source: Hum Vaccin Immunother. 2021 Apr 13;17(9):3230–8. doi: 10.1080/21645515.2021.1904758 (PMC8381844; doi:10.1080/21645515.2021.1904758)
Supplement: Supplemental Material [file KHVI_A_1904758_SM7795.docx]

**Supplementary table 1.** Number and percentage of strains covered in MATS and gMATS by 0, 1, 2, or 3 4CMenB vaccine antigens stratified by year of sample collection, state/territory, and patient’s age group

|  | N | MATS coverage, number (%) of strains | | | |  | gMATS coverage, number (%) of strains | | | | |
| --- | --- | --- | --- | --- | --- | --- | --- | --- | --- | --- | --- |
|  |  | 0 antigens | 1 antigen | 2 antigens | 3 antigens |  | 0 antigens | 1 antigen | 2 antigens | 3 antigens | Unpredictable |
| By year | | | | | | | | | | | |
| 2007 | 108 | 32 (29.6) | 37 (34.3) | 29 (26.9) | 10 (9.3) |  | 17 (15.7) | 40 (37.0) | 33 (30.6) | 7 (6.5) | 11 (10.2) |
| 2008 | 119 | 22 (18.5) | 45 (37.8) | 31 (26.1) | 21 (17.6) |  | 13 (10.9) | 34 (28.6) | 38 (31.9) | 19 (16.0) | 15 (12.6) |
| 2009 | 103 | 30 (29.1) | 40 (38.8) | 18 (17.5) | 15 (14.6) |  | 16 (15.5) | 42 (40.8) | 18 (17.5) | 18 (17.5) | 9 (8.7) |
| 2010 | 93 | 21 (22.6) | 40 (43.0) | 22 (23.7) | 10 (10.8) |  | 6 (6.5) | 36 (38.7) | 24 (25.8) | 16 (17.2) | 11 (11.9) |
| 2011 | 97 | 27 (27.8) | 35 (36.1) | 23 (23.7) | 12 (12.4) |  | 16 (16.5) | 30 (30.9) | 22 (22.7) | 13 (13.4) | 16 (13.4) |
| By state/territory | | | | | | | | | | | |
| NSW/ACT | 156 | 44 (28.2) | 65 (41.7) | 33 (21.2) | 14 (9.0) |  | 23 (14.7) | 61 (39.1) | 34 (21.8) | 17 (10.9) | 21 (14.5) |
| NT | 4 | 3 (75.0) | 1 (25.0) | 0 | 0 |  | 1 (25.0) | 1 (25.0) | 1 (25.0) | 0 | 1 (25.0) |
| Qld | 141 | 21 (14.9) | 60 (42.6) | 43 (30.5) | 17 (12.1) |  | 14 (9.9) | 54 (38.3) | 47 (33.3) | 18 (12.8) | 8 (5.7) |
| SA | 40 | 4 (10.0) | 6 (15.0) | 10 (25.0) | 20 (50.0) |  | 2 (5.0) | 7 (17.5) | 10 (25.0) | 20 (50.0) | 1 (2.5) |
| Tas | 9 | 4 (44.4) | 5 (55.6) | 0 | 0 |  | 3 (33.3) | 6 (66.7) | 0 | 0 | 0 |
| Vic | 118 | 31 (26.3) | 45 (38.1) | 29 (24.6) | 13 (11.0) |  | 11 (9.3) | 39 (33.1) | 32 (27.1) | 13 (11.0) | 23 (19.5) |
| WA | 52 | 25 (48.1) | 16 (30.8) | 8 (15.4) | 3 (5.8) |  | 14 (26.9) | 14 (26.9) | 11 (21.2) | 5 (9.6) | 8 (15.4) |
| By age group* | | | | | | | | | | | |
| <1 year | 75 | 27 (36.0) | 27 (36.0) | 17 (22.7) | 4 (5.3) |  | 16 (21.3) | 27 (36.0) | 13 (17.3) | 4 (5.3) | 15 (20.0) |
| 1–<2 years | 28 | 8 (28.6) | 10 (35.7) | 6 (21.4) | 4 (14.3) |  | 4 (14.3) | 11 (39.3) | 2 (7.1) | 6 (21.4) | 5 (17.9) |
| 2–<5 years | 30 | 5 (16.7) | 11 (36.7) | 10 (33.3) | 4 (13.3) |  | 0 | 11 (36.7) | 15 (50.0) | 3 (10.0) | 1 (3.3) |
| 5–29 years | 118 | 23 (19.5) | 50 (42.4) | 33 (28.0) | 12 (10.2) |  | 11 (9.3) | 40 (33.9) | 42 (35.6) | 15 (12.7) | 10 (8.4) |
| >29 years | 68 | 17 (25.0) | 28 (41.2) | 14 (20.6) | 9 (13.2) |  | 11 (16.2) | 23 (33.8) | 18 (26.5) | 8 (11.8) | 8 (11.8) |

MATS, meningococcal antigen typing system; gMATS, genetic MATS; 4CMenB, 4-component meningococcal serogroup B vaccine; N, number of tested isolates; NSW, New South Wales; ACT, Australian Capital Territory; NT, Northern Territory; Qld, Queensland; SA, Southern Australia; Tas, Tasmania; WA, Western Australia; Vic, Victoria.

Note: * The age of the patient from whom the sample was collected was only documented for 319 of the total 520 strains.
